# Aptamer-Based Triple Serum Fluorescence Intensity Assay: A Novel and Feasible Method for the Clinical Diagnosis of Primary Hepatic Carcinoma

**DOI:** 10.3389/fonc.2022.897775

**Published:** 2022-06-07

**Authors:** Jian-Ming Zhou, Fang-Li Han, Hong-Li Zhang, Ying Sun, Zi-Hua Li, Ting Wang, Kun-He Zhang

**Affiliations:** Department of Gastroenterology, Jiangxi Institute of Gastroenterology and Hepatology, The First Affiliated Hospital of Nanchang University, Nanchang, China

**Keywords:** aptamer, diagnosis, triple serum fluorescence, serum, primary hepatic carcinoma

## Abstract

**Background and Aims:**

Aptamers are artificial ligands that bind to biological targets with high specificity and affinity. We previously selected a group of aptamers against the serum of primary hepatic carcinoma (PHC) *via* systematic evolution of ligands by exponential and enrichment (SELEX) method, and some of the aptamers were valuable for PHC diagnosis in polyacrylamide gel electrophoresis (PAGE) analysis. Here, we used aptamers to develop a novel method suitable for the clinical diagnosis of PHC.

**Methods:**

The intensities of serum autofluorescence, cell-free DNA (cfDNA)-related fluorescence and aptamer-related fluorescence, named the aptamer-based triple serum fluorescence intensity (ATSFI), were sequentially measured at 8 °C and 37 °C in one tube by using a real-time polymerase chain reaction (PCR) system as a fluorimeter in patients with PHC (n=346) or liver cirrhosis (n=321). The diagnostic performances of ATSFI indicators alone and in combination were evaluated by area under the receiver operator characteristic curve (AUROC), and the underlying clinical mechanisms were analyzed by bivariate correlation.

**Results:**

The measurement of ATSFI was high throughput, rapid, convenient, and low cost. The aptamer-related fluorescence indicator SEA-SE37 was the most valuable for PHC diagnosis among all fluorescence indicators and superior to alpha-fetoprotein (AFP) (AUROC 0.879 vs. 0.836). The logistic model of ATSFI indicators exhibited excellent diagnostic performance for PHC, including AFP-negative, early and small PHCs, with AUROCs of 0.935-0.950 and accuracies of 86.8-88.3%. The diagnostic performance was further improved when ATSFI indicators were combined with AFP, with AUROCs of approximately 0.95 and accuracies of approximately 90%, suggesting ATSFI was independent of but complementary to AFP in PHC diagnosis. ATSFI models were highly valuable in clinical decision-making. The aptamer-related fluorescence intensity was generally independent of the clinicopathological characteristics of PHC but correlated with laboratory characteristics of PHC serum.

**Conclusions:**

The ATSFI assay is a novel, robust and feasible method for the clinical diagnosis of PHC.

## 1 Introduction

According to global cancer statistics in 2020, there are estimated 905,677 new cases and 830,180 deaths from liver cancer, ranking seventh and second for cancer incidence and death, respectively, and most of these cases occur in East Asia (1). Early diagnosis and curative treatment are crucial for improving the prognosis of patients with primary hepatic carcinoma (PHC) (2).

Aptamers are artificial nucleic acid ligands of biological molecules that are selected *via* the systematic evolution of ligands by exponential enrichment (SELEX) method (3, 4). Aptamers are similar to antibodies in function but superior to antibodies in application and thus promising in medical diagnosis (5). By using aptamer-based fluorescence spectroscopy, Wu et al. (6) developed a highly sensitive and specific method for the detection of non-small cell lung cancer cells in human serum and pleural effusion, with an area under the receiver operator characteristic curve (AUROC) of 0.974 for the early diagnosis of lung cancer.

Serum, the largest reservoir of disease biomarkers, provides rich information on disease occurrence and development (7) and therefore is a valuable complex target for selecting aptamers against disease biomarkers. Gold et al. (8) selected a group of aptamers against serum and then developed an aptamer chip that could detect 813 serum proteins with high throughput, and they found 12 proteins related to lung cancer from them and established a valuable discriminant model for lung cancer diagnosis, including early lung cancer (9).

Previously, we developed a universal method for the selection of serum aptamers and generated a group of aptamers against PHC serum (10). Briefly, the sera from patients with hepatocellular carcinoma (HCC) or intrahepatic cholangiocarcinoma (ICC) were pooled and used as targets for positive selection, and sera from normal controls were pooled and used as targets for negative selection. The pooled serum was incubated with the random single-stranded oligonucleotide library (artificially synthesized) and then the free oligonucleotide sequences (in negative selection) or bound oligonucleotide sequences (in positive selection) were separated and amplified for the next round of selection. The procedure (incubation, separation and amplification) was iterated to select and enrich the oligonucleotide sequences capable of binding to specific molecules (biomarkers) of PHC serum. After three rounds of negative selection followed by nine rounds of positive selection, the bound sequences of the last round of selection were cloned and sequenced, and finally we obtained a group of aptamers that were able to specifically bind to the serum biomarkers of PHC and to be used in the detection of PHC.

Based on analyzing the binding bands of aptamers to serum targets *via* polyacrylamide gel electrophoresis (PAGE), we found that some of these aptamers exhibited high diagnostic performance for PHC, with AUROCs superior to that of AFP (10). However, the PAGE-based procedure for the diagnostic evaluation of aptamers is low-throughput, laborious, and time-consuming, although it has the advantages of visibility and low cost. Fluorescence intensity is a frequent and simple method for the detection of aptamer-target binding (11, 12). A label-free fluorescence-based aptamer sensor could detect low abundance of exosomes derived from cancer cells in human blood with high sensitivity and specificity (13). Therefore, a fluorescence-based method to detect the binding of aptamers to serum targets may be an ideal alternative for the PAGE-based method.

Serum autofluorescence and cell-free DNA (cfDNA)-related fluorescence intensities are correlated with tumors (14, 15). In our previous work, by using a conventional real-time PCR system as a fluorimeter, we conveniently detected the autofluorescence and cfDNA-related fluorescence intensities of serum specimens, and by combining them, we developed a simple and high-throughput diagnostic method for PHC, but the method was not robust in differentiating PHC from liver cirrhosis (LC) (16). We speculated that using the same method to additionally detect the serum fluorescence intensity of the aptamer against PHC serum and combining it with the autofluorescence and cfDNA-related fluorescence intensities (together named aptamer-based triple serum fluorescence intensities, ATSFI) could yield robust diagnostic performance in differentiating PHC from LC.

Therefore, in the present study, we used a real-time PCR system to sequentially detect ATSFI in patients with PHC or LC in one tube and combined the indicators of ATSFI to develop a novel method with robustness, convenience and clinical potential for the diagnosis of PHC.

## 2 Materials and Methods

### 2.1 Collection of Serum Specimens and Clinical Data

**Figure 1:**
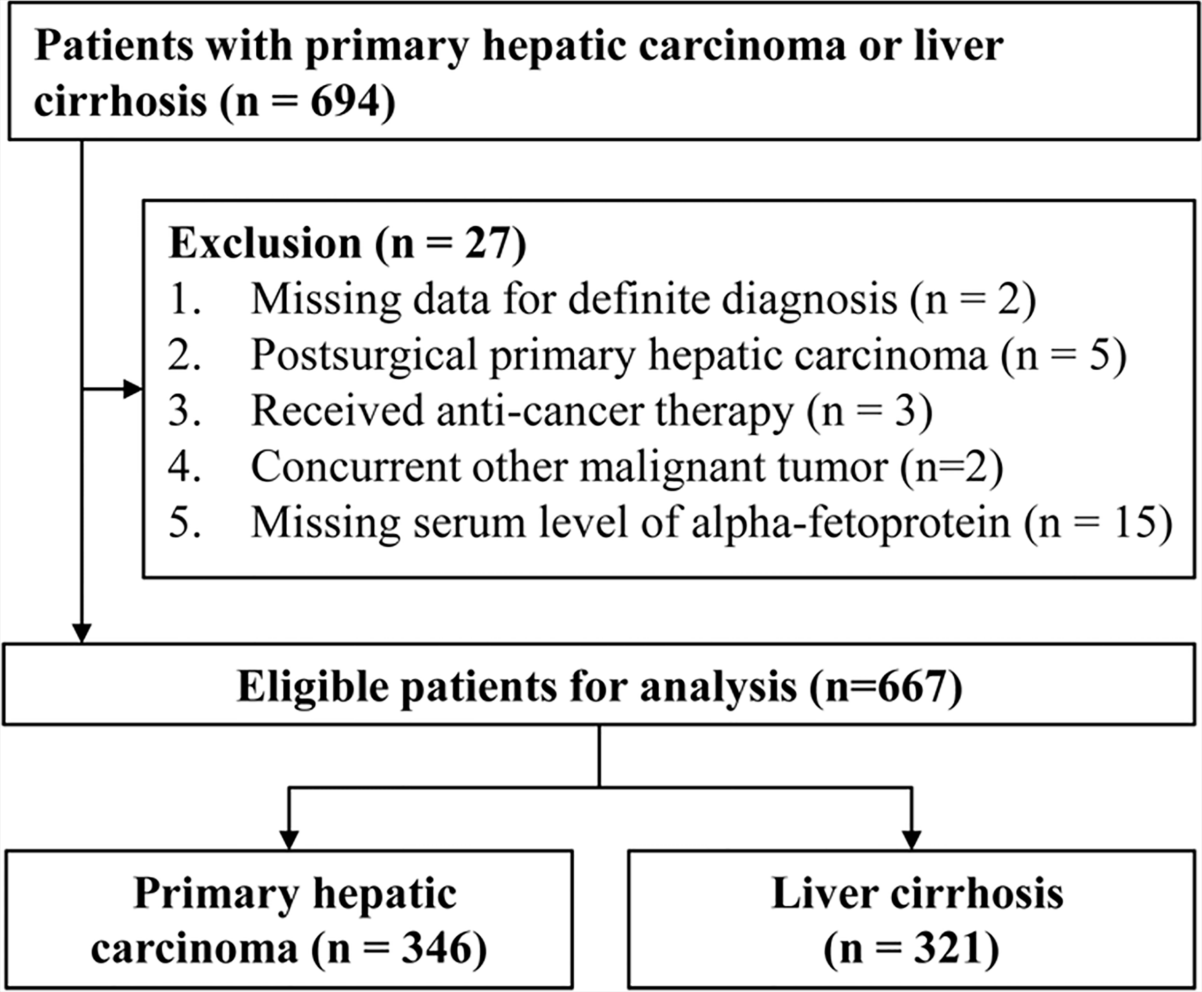
The flowchart of patient collection.

Leftover serum specimens (initially drawn for routine laboratory tests) and clinical data of hospitalized patients with PHC or LC before treatment were collected from the First Affiliated Hospital of Nanchang University from November 2014 to August 2020. Serum specimens were refrigerated at -80°C. The collected clinical data included age, sex, image examination, pathology, and blood laboratory tests (cell analysis, biochemistry, and coagulation and tumor markers). The diagnostic criteria of PHC and LC were the same as in our previous research (16). Patients with hepatocellular carcinoma (HCC) were staged according to the Barcelona Clinic Liver Cancer (BCLC) staging system (17). All PHC patients were staged according to the eighth version of the Tumour, Node, Metastases (TNM) staging system (18). This study was approved by The Ethics Committee on Medical Research of the First Affiliated Hospital of Nanchang University. The flowchart of patient collection is shown in **Figure 1**.

### 2.2 Measurement of Aptamer-Based Triple Serum Fluorescence Intensities

**Figure 2:**
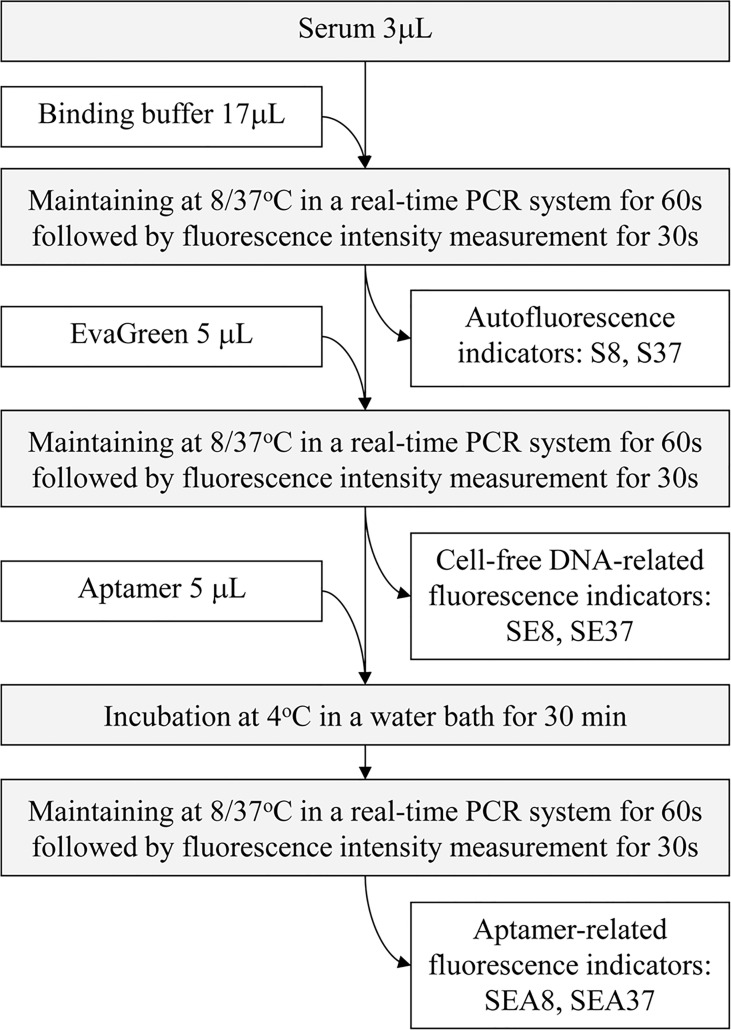
Diagram of the measurement of aptamer-based triple serum fluorescence intensities. S8, S37, SE8, SE37, SEA8 and SEA37: the names of 6 original fluorescence indicators, in which the abbreviations indicate serum (S), EvaGreen (E), aptamer (A), 8°C (8) or 37°C (37).

The ATSFIs were sequentially measured in one tube, in which a StepOne Plus™ real-time PCR system (Applied Biosystem, USA) was used as a fluorimeter. The real-time PCR system is capable of sequentially and cyclically implementing PCR reactions and fluorescent quantifications of amplification nucleic acid products at a set temperature and time, enabling high-throughput detection of genes. In the present study, the system was served as a fully automated fluorimeter to sequentially measure the fluorescence intensities at specific temperature and time. In the measurement, the amounts of serum, nucleic acid dye EvaGreen and aptamers and detection temperature should be optimized to obtain the best results for PHC diagnosis. Previously, we determined the optimal serum amount and temperature, that is, 3 µL serum volume at 8°C and 37°C (16); therefore, we only optimized the amounts of EvaGreen and aptamer in the present study. During the optimization, pooled sera of PHC and LC (40 randomly selected serum specimens mixed in the same volume) were first used to determine the best experimental conditions, and then individual serum specimens were used to validate the best conditions. The diagram of ATSFI measurement is shown in **Figure 2**.

#### 2.2.1 Optimization of the Optimal Amount of EvaGreen by Pooled Sera

Three microlitres of pooled sera of PHC or LC were mixed with 17 µL binding buffer (20 mM HEPES, 5 mM KCl, 1 mM CaCl_2_, 120 mM NaCl, 1 mM MgCl_2_, pH 7.35) and incubated in a real-time PCR system at 8°C or 37°C for 60 s followed by detection of fluorescence intensity for 30 s to obtain serum autofluorescence indicators S8 and S37. Then, 5 µL EvaGreen (Biotium, USA) at various concentrations (2×, 4×, 8×, and 16×) was added in proper order to the tubes and incubated in the real-time PCR system at 8°C or 37 °C for 60 s followed by detection of fluorescence intensity for 30 s to obtain the cfDNA-related fluorescence indictors SE8 or SE37. The test was performed in three duplicate tubes. The fluorescence intensity change of each tube after adding EvaGreen was calculated and presented as ratio indicators SE/S8 (SE8/S8) or SE/S37 (SE37/S37). The average ratios of these two ratio indicators in PHC to LC at each EvaGreen point were calculated, and the point with the largest difference was selected as the optimal EvaGreen concentration.

#### 2.2.2 Optimization of the Amount of Aptamer by Pooled Sera

In these tubes above, 5 µL of denatured aptamer AP-HCS-9-90 at different concentrations (0.02, 0.03, 0.04, 0.05, 0.06, 0.07 and 0.08 pmol/µL) was added in proper order, incubated at 4°C for 30 min, transferred to the PCR system and maintained at 8°C or 37°C for 60 s followed by the measurement of fluorescence intensity for 30 s to obtain the aptamer-related fluorescence indictors SEA8 or SEA37. The test was performed in three duplicate tubes. The fluorescence intensity change of each tube after adding aptamer was calculated and presented as ratio indicators SEA/SE8 (SEA8/SE8) or SEA/SE37 (SEA37/SE37). The average ratios of PHC to LC of these two indicators at each aptamer point were calculated, and the point with the largest difference was selected as the optimal aptamer concentration.

#### 2.2.3 Optimization of the Amount of Aptamers by Individual Serum Specimens

Under the optimal conditions determined above, individual serum specimens of PHC and LC (n=32 each) were used instead of the pooled sera to measure the ATSFIs. The serum autofluorescence and cfDNA-related fluorescence intensities were measured as described above, and the aptamer-related fluorescence intensity was measured at three aptamer concentrations. For each aptamer concentration point, six original ATSFI indicators (S8, S37, SE8, SE37, SEA8 and SEA37) were directly measured in each specimen, and eight derived ATSFI indicators (SE-S8, SE-S37, SEA-SE8, SEA-SE37, SE/S8, SE/S37, SEA/SE8 and SEA/SE37) were calculated by subtraction or division between the six original indicators. These fluorescence intensity indicators were named by a combination of abbreviations: S (Serum), E (EvaGreen), A (Aptamer), 8 (8°C) and 37 (37°C) and indicated the serum fluorescence intensity measured under a specific condition; for example, SEA-SE37 indicates the fluorescence intensity difference of serum incubated with EvaGreen in the presence and absence of aptamer at 37°C. These original and derived indicators were used to generate receiver operator characteristic (ROC) curves for differentiating PHC and LC, and the point with the maximum AUROC was selected as the optimal aptamer concentration for subsequent analysis.

### 2.3 Measurement of ATSFIs in Clinical Serum Specimens

Using the optimal condition determined in the individual serum specimens, the measurement of ATSFIs was performed in clinical serum specimens, and six original and eight derived ATSFI indicators were obtained.

### 2.4 Diagnostic Evaluation of the ATSFI for Primary Hepatic Carcinoma

The diagnostic value of ATSFI indicators alone and in combination was evaluated by using AUROC and sensitivity, specificity, accuracy, positive and negative predictive values, and positive and negative likelihood ratios. For the combination analysis, the patients were randomly divided into a training set and a testing set at an appropriate ratio of 6:4. The ATSFI indicators of the training set were used as independent variables to develop a diagnostic model for PHC by using binary logistic regression analysis, and the model was validated using the data of the testing set. The goodness of fit of the model was evaluated by the Hosmer-Lemeshow test and calibration curve. The clinical utility of ATSFI was evaluated by decision curve analysis (19, 20). The calibration curves and decision curves were plotted using R software.

### 2.5 Statistical Analysis

Measurement data are presented as the mean ± standard deviation (SD) if normally distributed or median (M) (interquartile range, IQR) if not normally distributed. Enumeration data are presented as counts and percentages. Statistical tests for the comparison of variables between groups were dependent on the specific situations and given in the text in detail. P<0.05 was considered a significant difference. All statistical analyses were performed by SPSS 22.0 (IBM, NY, USA).

## 3 Results

### 3.1 Demographic and Clinical Data of Patients

A total of 667 eligible patients were entered into this study, including 346 cases of PHC and 321 cases of LC. The demographic and clinical characteristics of these patients are shown in **Table 1**.

**Table 1:** The demographic and clinical characteristics of patients.

	PHC (n = 346)	LC (n = 321)	P
**Age (Mean ± SD, years)**	55.7 ± 12.1	52.4 ± 11.5	<0.001^a^
**Sex [n (%)]**			
Male	276 (79.8)	237 (73.8)	0.069^b^
Female	70 (20.2)	84 (26.2)	
**Etiology [n (%)]**			
HBV	272 (78.6)	225 (70.1)	<0.001^c^
Non-HBV	21 (6.1)	62 (19.3)	
Unknown	53 (15.3)	34 (10.6)	
**Blood cell analyses**			
WBC (×10^9^/L) [M (IQR)]	5.4 (4.1-6.8)	3.3 (2.5-4.7)	<0.001^d^
RBC (×10^12^/L) [M (IQR)]	4.3 (3.8-4.8)	3.7 (3.0-4.2)	<0.001^d^
HB (g/L) [M (IQR)]	133.0 (120.0-147.0)	103.0 (84.0-122.0)	<0.001^d^
PLT (×10^9^/L) [M (IQR)]	172.0 (111.5-232.5)	66.0 (44.0-110.5)	<0.001^d^
**Hepatic function test**			
ALT (U/L) [M (IQR)]	32.0 (22.0-55.3)	26.0 (18.0-39.0)	<0.001^d^
AST (U/L) [M (IQR)]	42.5 (28.0-80.3)	39.0 (29.0-60.0)	0.092 ^d^
TBIL (μmol/L) [M (IQR)]	13.9 (9.55-21.2)	19.1 (12.4-30.2)	<0.001^d^
DBIL (μmol/L) [M (IQR)]	4.4 (2.9-7.5)	8.7 (5.2-13.9)	<0.001^d^
GGT (U/L) [M (IQR)]	83.0 (41.8-174.5)	33.0 (18.0-60.0)	<0.001^d^
ALP (U/L) (Mean ± SD)	160.7 ± 150.5	110.6 ± 58.3	<0.001^a^
TP (g/L) (Mean ± SD)	68.8 ± 7.2	62.7 ± 8.0	<0.001^a^
ALB (g/L) (Mean ± SD)	38.8 ± 5.8	33.5 ± 5.8	<0.001^a^
GLB (g/L) (Mean ± SD)	30.0 ± 6.4	29.2 ± 7.2	0.134 ^a^
**Child-Pugh grade [n (%)]**			
A	292 (84.4)	197 (61.4)	<0.001^b^
B	42 (12.1)	70 (21.8)	
C	12 (3.5)	54 (16.8)	
**Alpha-fetoprotein**			
Serum level (ng/mL) [M (IQR)]	42.6 (4.5-1210.0)	2.2 (1.5-4.4)	<0.001^d^
Positive (>= 20 ng/mL) [n (%)]	190 (54.9)	24 (7.5)	<0.001^b^
Negative (< 20 ng/mL) [n (%)]	156 (45.1)	297 (92.5)	
**Tumor size**		NA	NA
Diameter (cm) (mean ± SD)	6.8 ± 3.8		
Small (<= 3 cm) [n (%)]	65 (18.8)		
Large (> 3 cm) [n (%)]	281 (81.2)		
**Tumor type**		NA	NA
Hepatocellular carcinoma [n (%)]	307 (88.7)		
Intrahepatic cholangiocarcinoma [n (%)]	39 (11.3)		
**BCLC stage for HCC [n (%)]**		NA	NA
A	142 (46.3)		
B	58 (18.9)		
C	102 (33.2)		
D	5 (1.6)		
**TNM stage [n (%)]**		NA	NA
I	143 (41.3)		
II	33 (9.5)		
III	93 (26.9)		
IV	77 (22.3)		

a Student’s t-test.

b Pearson Chi-squared test.

c Fisher’s exact test.

d Mann-Whitney U test.

PHC: primary hepatic carcinoma; LC, liver cirrhosis; HBV, hepatitis B virus; WBC, white blood cell; RBC, red blood cell; HB, hemoglobin; PLT, Platelet count; ALT, alanine aminotransaminase; AST, aspartate aminotransaminase; TBIL, total serum bilirubin; DBIL, direct serum bilirubin; GGT, glutamyl transferase; ALP, alkaline phosphatase; TP, total serum protein; ALB, serum albumin; GLB, serum gamma-globulins; NA, not applicable; BCLC, Barcelona Clinic Liver Cancer; HCC, hepatocellular carcinoma; TNM, Tumor, Node, Metastases.

### 3.2 Optimal Conditions for the Measurement of ATSFIs

**Figure 3:**
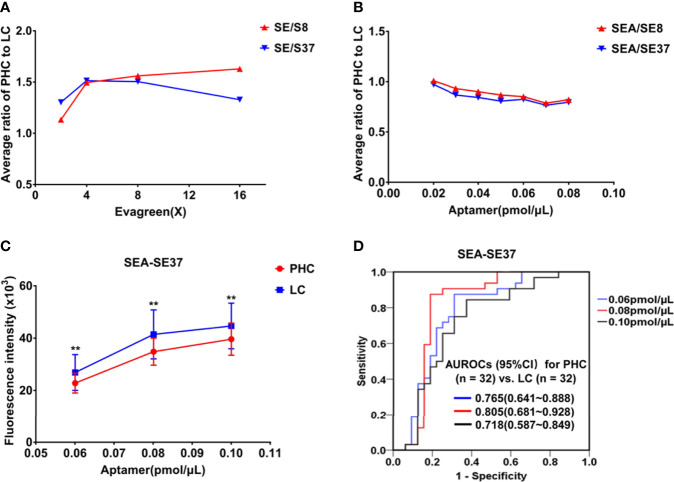
Optimization of measurement conditions for aptamer-based triple serum fluorescence intensities. **(A)** The fluorescence indicator ratios of PHC to LC in a series of EvaGreen concentrations in pooled sera (n = 3). **(B)** Fluorescence indicator ratios of PHC to LC at a series of aptamer AP-HCS-9-90 concentrations and 8× EvaGreen in pooled sera (n = 3). **(C)** The fluorescence indicator SEA-SE37 of serum specimens (n = 32, PHC and LC each) at various aptamer AP-HCS-9-90 concentrations and 8× EvaGreen. **P < 0.01. **(D)** Receiver operator characteristic curves of SEA-SE37 for the diagnosis of PHC at various aptamer AP-HCS-9-90 concentrations. Abbreviations in the fluorescence indicators: S, serum; E, EvaGreen; A, aptamer; 8, 8°C; 37, 37°C; SE/S8 or SE/S37: the fluorescence intensity ratio of serum reacted with EvaGreen to original serum at 8°C or 37°C; SEA-SE8 or SEA-SE37, the fluorescence intensity difference of serum incubated with 8× EvaGreen and aptamer and serum reacted with 8× EvaGreen at 8°C or 37°C. PHC, primary hepatic carcinoma; LC, liver cirrhosis; AUROC, area under the receiver operator characteristic curve; CI, confidence interval.

The measurement conditions of ATSFI were first optimized by using pooled sera, and the results showed that the ratios of PHC to LC became stable in ATSFI indicators SE/S8 and SE/S37 at 8× EvaGreen (**Figure 3A**) and SEA/SE8 and SEA/SE37 at 0.08 pmol/μL aptamer (**Figure 3B**). Thus, 8× EvaGreen and 0.08 pmol/μL aptamers were selected as the optimal EvaGreen and aptamer concentrations, respectively. Under the optimal conditions, we performed ATSFI measurements in individual serum specimens of PHC and LC (n=32, each), and the results showed that the indicator SEA-SE37 presented the largest AUROC among all ATSFI indicators for the diagnosis of PHC and that among the three aptamer concentration points, SEA-SE37 at the point of 0.08 pmol/µL had the largest difference of fluorescence intensity between PHC and LC (**Figure 3C**) and the highest AUROC for the diagnosis of PHC (**Figure 3D**), the same as the result in the optimization of pooled sera. Therefore, 0.08 pmol/µL was selected as the best concentration of aptamer AP-HCS-9-90 in the ATSFI measurement.

### 3.3 ATSFIs of Clinical Specimens and Their Diagnostic Value

The ATSFIs of aptamer AP-HCS-9-90 were measured in the 667 cases, and their diagnostic values for PHC were evaluated (**Table 2**). The serum fluorescence intensities increased when EvaGreen and aptamer were added. The fluorescence indicators SEA-SE8 and SEA-SE37 had the maximum AUROCs among all fluorescence indicators.

**Table 2:** Aptamer-based triple serum fluorescence intensities and their AUROCs for the diagnosis of PHC.

Indicator	Fluorescence intensity [×10^3^, M (IQR)]		P*	AUROC (95%CI)
	PHC (n = 346)	LC (n = 321)		
S8	20.0 (14.8-30.8)	24.0 (17.4-35.2)	<0.001	0.578 (0.535-0.622)
SE8	80.5 (65.1-101.5)	67.6 (58.3-84.2)	<0.001	0.628 (0.585-0.671)
SEA8	139.9 (124.1-157.6)	145.4 (135.3-161.4)	<0.001	0.609 (0.567-0.652)
SE-S8	54.2 (42.0-72.1)	41.8 (33.5-51.0)	<0.001	0.686 (0.645-0.727)
SEA-SE8	58.9 (51.1-68.9)	78.5 (70.9-83.4)	<0.001	0.814 (0.780-0.847)
SE/S8^#^	3.8 (2.5-5.3)	2.8 (2.1-4.1)	<0.001	0.630 (0.588-0.672)
SEA/SE8^#^	1.8 (1.5-2.0)	2.1 (1.9-2.4)	<0.001	0.738 (0.700-0.775)
S37	10.9 (7.4-18.2)	13.9 (9.6-20.4)	<0.001	0.595 (0.552-0.638)
SE37	48.7 (39.5-61.2)	40.9 (35.1-50.8)	<0.001	0.636 (0.594-0.679)
SEA37	71.5 (62.4-81.3)	78.7 (71.4-87.8)	<0.001	0.658 (0.617-0.699)
SE-S37	35.2 (27.2-46.5)	25.5 (20.7-32.2)	<0.001	0.703 (0.663-0.744)
SEA-SE37	23.3 (17.1-28.7)	37.8 (31.8-41.1)	<0.001	0.879 (0.853-0.905)
SE/S37^#^	4.2 (2.7-6.2)	2.9 (2.2-4.5)	<0.001	0.634 (0.592-0.676)
SEA/SE37^#^	1.5 (1.3-1.7)	1.9 (1.7-2.1)	<0.001	0.801 (0.767-0.834)

*Mann-Whitney U test. ^#^Original value, not expressed as “×10^3^”. M, median; IRQ, interquartile range; AUROC, area under the receiver operator characteristic curve; PHC, primary hepatic carcinoma; LC, liver cirrhosis. Abbreviations in fluorescence indicators: S, serum; E, EvaGreen; A, aptamer; 8, 8°C; 37, 37°C. A fluorescence indicator indicates the serum fluorescence intensity under a specific condition; for example, SEA-SE37 indicates the fluorescence intensity difference of serum incubated with EvaGreen in the presence and absence of aptamer at 37°C.

### 3.4 Establishment of a Diagnostic Model Based on ATSFI

**Figure 4:**
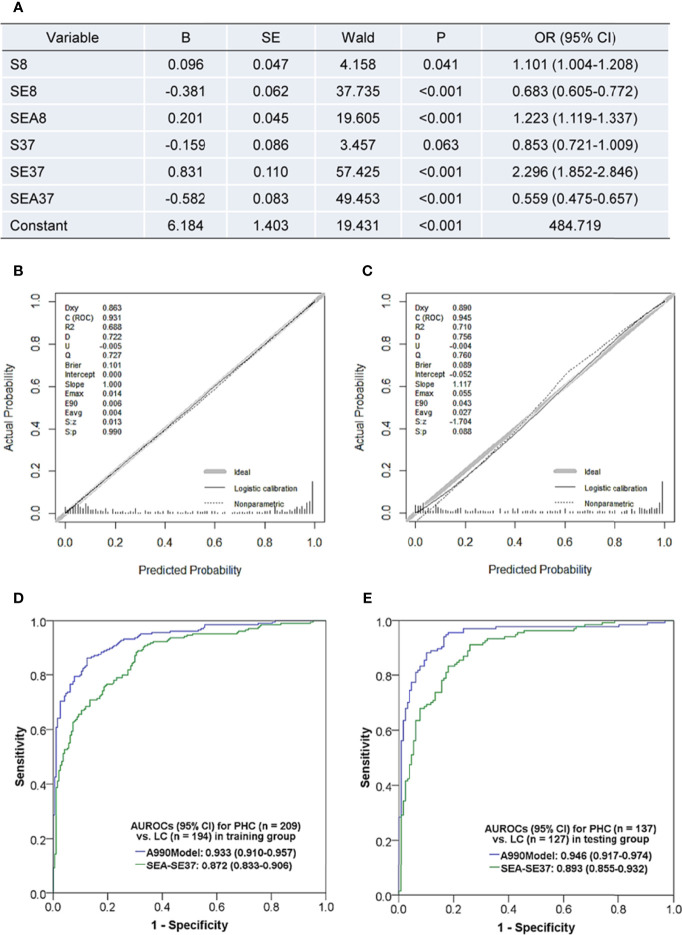
Diagnostic model based on the triple serum fluorescence indicators of aptamer AP-HCS-9-90 and its evaluation. **(A)** Variables and their information in the diagnostic model. **(B, C)** Calibration curves of the model in the training group **(B)** and testing group **(C)**. **(D, E)** Receiver operator characteristic curves of the model in the training group **(D)** and testing group **(E)**. Abbreviations in fluorescence indicators: S, serum; E, EvaGreen; A, aptamer; 8, 8°C; 37, 37°C. Fluorescence indicator indicates the serum fluorescence intensity under a specific condition; for example, SEA37 represents the serum fluorescence intensity at 37°C after incubation with EvaGreen and aptamer. B, regression coefficient; SE, standard error; OR, odds ratio; AUROC, the area under the receiver operator characteristic curve; CI, confidence interval; PHC, primary hepatic carcinoma; LC, liver cirrhosis; A990Model, the diagnostic model based on the six original fluorescence indicators of aptamer AP-HCS-9-90; SEN, sensitivity; SPE, specificity; ACC, accuracy; PPV, positive predictive value; NPV, negative predictive value; PLR, positive likelihood ratio; NLR, negative likelihood ratio.

The patients were randomly divided into a training set (PHC n = 209, LC n = 194) and a testing set (PHC n = 137, LC n = 127) by using the function “select cases” in SPSS software. Using six original ATSFI indicators of the training set as independent variables, a diagnostic model was established (**Figure 4A**):


Logit(P)=6.184+0.096×S8−0.381×SE8+0.201×SEA8−0.159×S37+0.831×SE37−0.582×SEA37

The model had a Nagelkerke R^2^ of 0.695 and was insignificant in Hosmer-Lemeshow test (P = 0.638). The calibration curves showed that the predicted probability was well in accordance with the observed probability in both the training and testing sets (**Figures 4B, C**), with very small maximum errors (Emax) and average errors (Eevg) for the training set (0.014 and 0.004, respectively) and testing set (0.055 and 0.027, respectively). The AUROCs of the model were more than 0.93 in both the training and testing sets (**Figures 4D, E**) and even better in the testing set. Additionally, the model exhibited much better diagnostic performance than SEA-SE37, the most valuable single indictor for PHC diagnosis. These results implicate that a valuable and stable model is established with six original indicators of ATSFI.

### 3.5 The Diagnostic Performances of ATSFI Indicators Alone and in Combination

**Figure 5:**
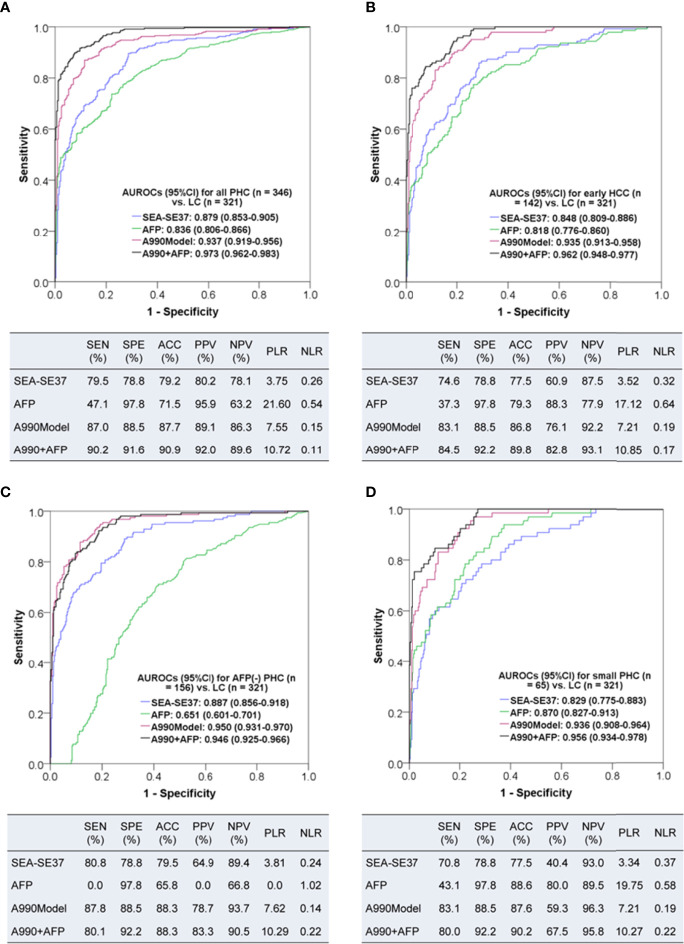
The diagnostic performances of triple serum fluorescence intensity of aptamer AP-HCS-9-90 alone and in combination for primary hepatic carcinoma. **(A–D)**: Diagnostic performances for all patients, early HCC (BCLC stage A), AFP-negative (< 20 ng/mL) PHC, and small (<= 3 cm) PHC, respectively. AUROC, area under the receiver operator characteristic curve; CI, confidence interval; PHC, primary hepatic carcinoma; LC, liver cirrhosis; HCC, hepatocellular carcinoma; SEA-SE37, the difference in fluorescence intensity at 37°C between the serum that was incubated with EvaGreen and aptamer and the serum that reacted with EvaGreen; AFP, alpha-fetoprotein; A990Model, the diagnostic model based on the six original fluorescence indicators of AP-HCS-9-90; A990+AFP, the diagnostic model based on the six original fluorescence indicators of AP-HCS-9-90 and AFP; SEN, sensitivity; SPE, specificity; ACC, accuracy; PPV, positive predictive value; NPV, negative predictive value; PLR, positive likelihood ratio; NLR, negative likelihood ratio.

To comprehensively evaluate the diagnostic value of ATSFI for PHC, the diagnostic performances of the best single indicator SEA-SE37, AFP, A990Model, and the combination of six original fluorescence indicators with AFP (A990+AFP) were evaluated and compared in all PHC, early HCC (BCLC stage A), AFP-negative PHC, and small PHC (**Figure 5**). We found that in all PHC and subtype groups of PHC, the SEA-SE37 had higher AUROCs than AFP (except in small PHC), the model derived from six original indicators had AUROCs of 0.935-0.950, much higher than AFP, and the diagnostic performances were further improved when the model was combined with AFP, with AUROCs of 0.946-0.973, sensitivities of 80.0-90.2%, specificities of 91.6-92.2%, and accuracies of 88.3-90.9%.

Additionally, we have separately analysed HCC and ICC, and the results showed that the AUROCs of the ATSFI model were 0.934 (95%CI 0.915-0.953) for distinguishing HCC from LC and 0.966 (95% CI 0.939-0.993) for distinguishing ICC from LC; the best single indicator remained SEA-SE37, with AUROCs of 0.877 (95%CI 0.850-0.904) for distinguishing HCC from LC and 0.892 (95%CI 0.837-0.947) for distinguishing ICC from LC. These results indicate good diagnostic value can be obtained in both of HCC and ICC.

### 3.6 The Clinical Utility of ATSFI Indicators Alone and in Combination

**Figure 6:**
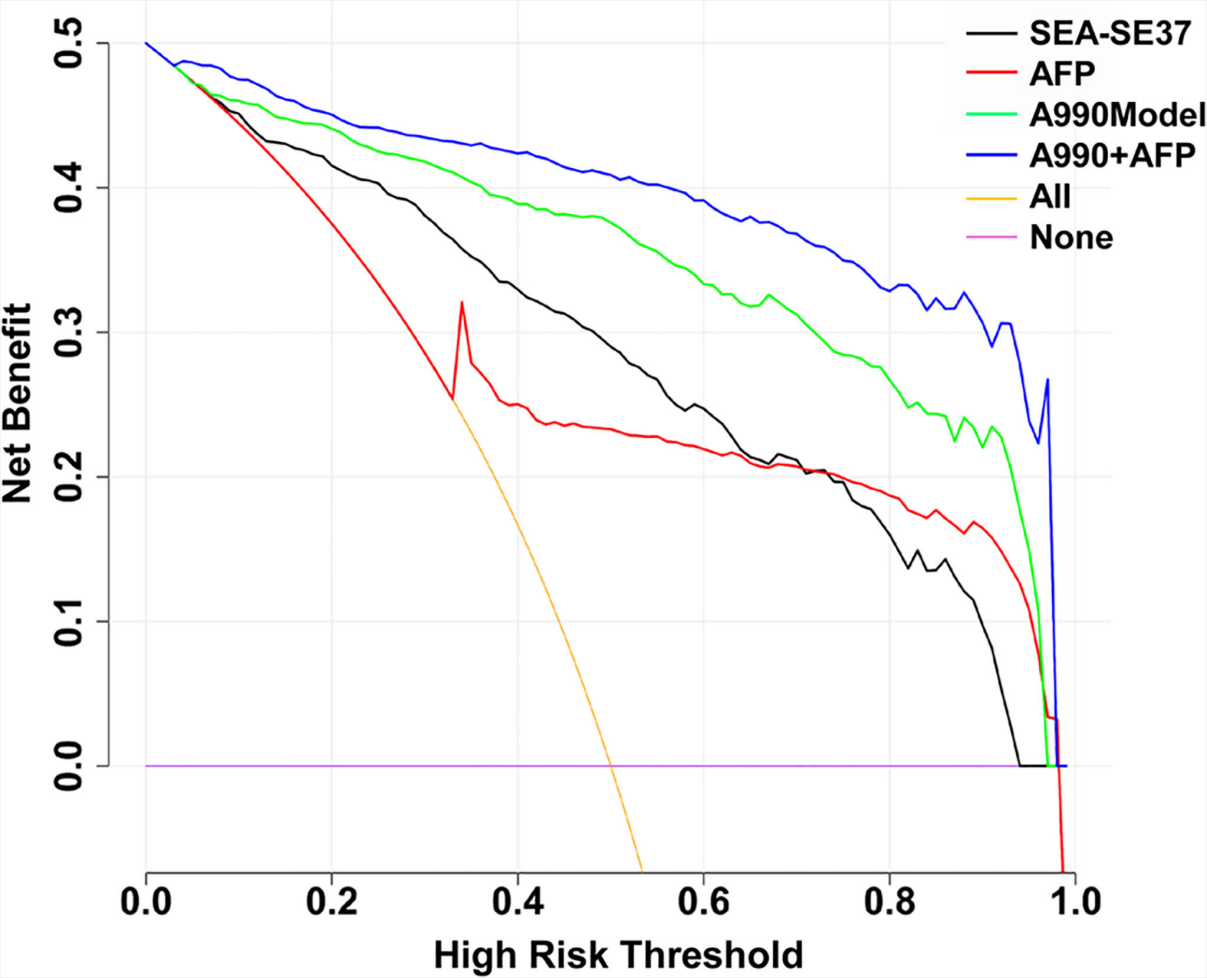
Decision curves of the aptamer-based triple serum fluorescence intensity indicators alone and in combination. SEA-SEA37: the difference in fluorescence intensity at 37°C between the serum that was incubated with EvaGreen and aptamer and the serum that reacted with EvaGreen. AFP: alpha-fetoprotein. A990Model: the diagnostic model established by the combination of triple serum fluorescence indicators (six original) of aptamer AP-HCS-9-90. A990+AFP: diagnostic model established by the combination of triple serum fluorescence indicators (six original) of aptamer AP-HCS-9-90 and AFP.

The decision curves of SEA-SE37, AFP, A990Model, and A990+AFP were plotted to evaluate the clinical utility of ATSFI (**Figure 6**). The results showed that A990+AFP had the largest net benefit, followed by A990Model, SEA-SE37, and AFP, indicating that according to the ATSFI alone or in combination with AFP, much higher net benefits could be obtained in deciding whether further diagnostic intervention is needed for identifying PHC.

### 3.7 Correlations of ATSFI Indicators With Blood Laboratory Indicators

To analyze the correlations of ATSFIs with the conventional blood laboratory test results, bivariate correlation analysis was performed between the fluorescence and blood indicators (**Table 3**). The results showed that serum autofluorescence (S8 and S37) was generally correlated with hepatic function, especially serum bilirubin, with a moderate correlation at 8°C, and the correlations weakened at 37°C. The cfDNA-related fluorescence indicators (SE8 and SE37) were very weakly correlated with some blood indicators, as were the aptamer-related fluorescence indicators (SEA8 and SEA37). However, the difference indicators of fluorescence intensity before and after adding aptamer (SEA-SE8 and SEA-SE37) showed wide and weak correlations with blood indicators.

**Table 3:** Correlations of aptamer-based triple serum fluorescence intensity indicators with laboratory blood indicators.

	Pearson’s correlation coefficient							
	S8	SE8	SEA8	SEA-SE8	S37	SE37	SEA37	SEA-SE37
**AFP**	0.006	0.026	-0.032	-0.142**	0.014	0.044	-0.052	-0.219**
**ALT**	0.230**	0.090*	0.036	-0.140**	0.196**	0.076*	0.019	-0.135**
**AST**	0.315**	0.111**	0.067	-0.118**	0.269**	0.104**	0.055	-0.118**
**TBIL**	0.511**	0.058	-0.020	-0.193**	0.324**	-0.013	-0.057	-0.099*
**DBIL**	0.496**	0.060	-0.017	-0.191**	0.311**	-0.008	-0.053	-0.100**
**GGT**	0.177**	0.082*	-0.014	-0.240**	0.126**	0.082*	-0.035	-0.270**
**ALP**	0.184**	0.059	-0.028	-0.214**	0.112**	0.053	-0.048	-0.231**
**TP**	-0.095*	0.051	-0.036	-0.214**	-0.075	0.057	-0.067	-0.284**
**ALB**	-0.245**	-0.047	-0.128**	-0.189**	-0.205**	-0.045	-0.155**	-0.245**
**GLB**	0.116**	0.103**	0.075	-0.081*	0.102**	0.108**	0.062	-0.112**
**AGR**	-0.198**	-0.094*	-0.120**	-0.049	-0.170**	-0.098*	-0.127**	-0.056
**Cr**	-0.006	-0.038	-0.019	0.051	-0.001	-0.030	-0.012	0.043
**BUN**	0.039	-0.053	-0.048	0.020	0.031	-0.056	-0.053	0.012
**TC**	-0.019	0.013	-0.095*	-0.258**	-0.029	0.015	-0.135**	-0.340**
**TG**	0.078*	0.000	-0.065	-0.155**	0.048	-0.009	-0.089*	-0.180**
**HDLC**	-0.332**	-0.094*	-0.074	0.061	-0.255**	-0.076	-0.062	0.038
**LDLC**	-0.100*	0.018	-0.059	-0.186**	-0.059	0.039	-0.091*	-0.297**
**WBC**	0.100**	0.139**	0.055	-0.218**	0.084*	0.149**	0.020	-0.303**
**RBC**	-0.154**	0.010	-0.033	-0.105**	-0.109**	0.015	-0.057	-0.164**
**Hb**	-0.137**	-0.063	-0.142**	-0.179**	-0.125**	-0.073	-0.186**	-0.250**
**PLT**	-0.083*	0.131**	0.010	-0.306**	-0.080*	0.148**	-0.033	-0.420**
**PT**	0.278**	0.005	0.030	0.059	0.203**	-0.032	0.030	0.145**
**APTT**	0.133**	0.016	0.093*	0.181**	0.117**	0.005	0.102**	0.219**
**INR**	0.300**	0.001	0.042	0.098*	0.225**	-0.031	0.045	0.175**

*P<0.05, **P<0.01. AFP, alpha-fetoprotein; ALT, alanine aminotransaminase; AST, aspartate aminotransaminase; TBIL, total serum bilirubin; DBIL, direct serum bilirubin; GGT, glutamyltransferase; ALP, alkaline phosphatase; TP, total serum protein; ALB, serum albumin; GLB, serum gamma-globulin; AGR, albumin and gamma-globulin ratio; Cr, creatinine; BUN, blood urea nitrogen; TC, total cholesterol; TG, triglyceride; HDLC, high-density lipoprotein cholesterol; LDHC, low-density lipoprotein cholesterol; WBC, white blood cell; RBC, red blood cell; Hb, hemoglobin; PLT, platelet; PT, prothrombin time; APTT, activated partial thrombin time; INR, international normalized ratio. Abbreviations in fluorescence indicators: S, serum; E, EvaGreen; A, aptamer; 8, 8°C; 37, 37°C. A fluorescence indicator indicates the serum fluorescence intensity under a specific condition; for example, SEA8 represents the serum fluorescence intensity at 8°C after incubation with EvaGreen and aptamer.

### 3.8 Associations of Aptamer-Related Fluorescence Indicators With Clinicopathological Characteristics of Primary Hepatic Carcinoma

**Figure 7:**
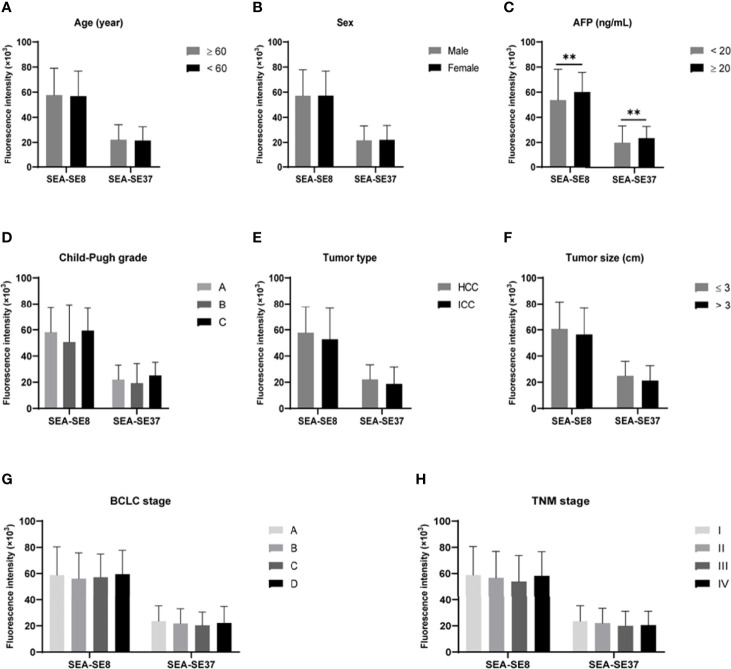
Comparisons of aptamer-related fluorescence indicators SEA-SE8 and SEA-SE37 between subgroups derived from various clinicopathological characteristics of primary hepatic carcinoma. **P < 0.01. **(A)** Subgroups of age. **(B)** Subgroups of sex. **(C)** Subgroups of AFP levels. **(D)** Subgroups of Child-Pugh grade. **(E)** Subgroups of tumor pathological type. **(F)** Subgroups of tumor size. **(G)** Subgroups of BCLC stage. **(H)** Subgroups of TNM stage. SEA-SE8 or SEA-SE37: the difference of fluorescence intensity at 8°C or 37°C measured after serum incubated with EvaGreen and aptamer and after serum reacted with EvaGreen. AFP, alpha-fetoprotein; BCLC, Barcelona Clinic Liver Cancer; TNM, Tumor, Node, Metastases.

The associations of aptamer-related fluorescence intensities with clinicopathological indictors of PHC were analyzed. The differences of SEA-SE8 and SEA-SE37 between subgroups of age, sex, AFP level, Child-Pugh grade, tumor type, tumor size, BCLC stage, and TNM stage were compared (**Figure 7**). The results showed that the two fluorescence indicators were not significant between the subgroups except AFP.

## 4 Discussion

In the present study, we successfully developed a novel and simple but robust method for the diagnosis of PHC. We sequentially measured the ATSFIs of PHC and LC in one tube by using a conventional real-time PCR system, evaluated the diagnostic value of ATSFIs for PHC, and analyzed the associations of ATSFIs with clinical and pathological characteristics. We found that some ATSFI indicators alone or in combination were capable of differentiating PHC (including early-stage HCC, small PHC and AFP-negative PHC) from LC with excellent performance, independence of but complementary to AFP, and good clinical utility and that aptamer-related fluorescence intensities were generally independent of the clinicopathological characteristics of PHC but correlated with multiple laboratory characteristics of PHC serum.

The ATSFI method has the advantages of high throughput, rapidity, convenience, and low cost. The original serum was directly used to measure autofluorescence followed by the measurements of cfDNA-related fluorescence intensity after adding EvaGreen and aptamer-related fluorescence intensity after adding aptamer and incubation. The ATSFIs could be measured sequentially in one tube within one hour, which not only made the measurement convenient, rapid and economical but also minimized measurement error. The real-time PCR system was used as a fluorimeter to measure the ATSFIs, which provided the advantages of high throughput in sample detection, precise control in detection temperatures, convenience in instrument accessibility, and “zero cost” in fluorescence detectors.

The ATSFI method exhibited high diagnostic performance for PHC. The cfDNA-related fluorescence indicators (SE-S8 and SE-S37) and aptamer-related fluorescence indicators (SEA-SE8 and SEA-SE37) were valuable in the differentiation of PHC from LC, particularly the indicator SEA-SE37, which exhibited a higher AUROC than AFP and was comparable to our previous PAGE-based method (10). The diagnostic model that incorporated the six original ATSFI indicators exhibited excellent diagnostic performances for PHC, including AFP-negative PHC, early-stage HCC and small PHC, with AUROCs greater than 0.93 and accuracies greater than 85%. Furthermore, the diagnostic value of the ATSFI in combination with AFP was further improved, even in AFP-negative PHC, with AUROCs up to approximately 0.95 and accuracies up to approximately 90%, which indicates that the ATSFIs are independent of but complementary to AFP in the diagnosis of PHC and therefore have good feasibility in clinical practice.

The ATSFI method is superior to our previous methods. Our previous diagnostic models that combined serum autofluorescence and cfDNA-related fluorescence intensities with conventional blood tests exhibited excellent diagnostic performances for the diagnosis of PHC (16, 21), comparable to the ATSFI method. However, the ATSFI model was developed with the six original fluorescence indicators from one test and without the combination of conventional blood tests, which does not need the collection of conventional blood test results and avoids the blood test results incomparable between medical centers due to difference of methods or reagents. Furthermore, the ATSFI method overcome the disadvantages of the low-throughput, laborious and time-consuming nature of our previous PAGE-based method that combined the grey indicators of two aptamers with AFP and exhibited diagnostic performance comparable to the ATSFI method (10). Therefore, the ATSFI method shows an obvious improvement in convenience and clinical feasibility compared with previous methods.

Decision curve analysis (DCA) has been widely utilized to evaluate the practical value of an indicator or predictive model in clinical decision-making (19, 20), which compares the net benefit of selective intervention according to the indicator or model with the net benefit of nonselective intervention (towards all or none), and greater net benefit than the all or none indicates that the indicator or model has better clinical utility. In the present study, DCA demonstrated that patients at a risk of PHC could obtain a much greater net benefit from diagnostic intervention to be decided according to the ATSFI compared with AFP, especially in combination (**Figure 6**). These results suggest that the ATSFI is valuable in clinical application.

Serum autofluorescence is produced by native fluorescent substances in the blood and has been reported to have potential in the diagnosis of liver cancer (22), but in the present study, serum autofluorescence indicators (S8 and S37) were not valuable for the diagnosis of PHC, although they were significantly lower in PHC than in LC. We observed a strong correlation of autofluorescence intensities with serum levels of both total and direct bilirubin in correlation analysis, which indicates that the autofluorescence is mainly from serum bilirubin. Since serum bilirubin is just an indicator for the impairment of liver function, it is rational for the serum autofluorescence indicators to be not significant in differentiating PHC from LC.

When the nucleic acid dye EvaGreen was added, the serum fluorescence intensity increased in both PHC and LC, but the corresponding fluorescence indicators (SE8 and SE37) showed weak diagnostic value in differentiating PHC and LC. However, the changes in fluorescence intensity (SE-S8 and SE-S37) exhibited significant diagnostic value due to the different increases between PHC and LC, indicating that compared with LC, PHC has higher levels of circulating cfDNA that bind to EvaGreen and emit stronger fluorescence. Circulating cfDNA is the fragment of DNA derived from the apoptosis and necrosis of cells, especially in patients with tumor (23). Circulating cfDNA has potential diagnostic value in liver cancer (24), but it is not robust enough to be used independently (25). Here, an interesting issue is whether HBV-DNA affects cfDNA levels and their fluorescence intensities? The patients in the present study were treating with nucleic acid analogues according to the guideline (26), and thus only 137 patients had measurable HBV-DNA levels from 2.52×10^2^ to 9.01×10^7^ IU/mL. We performed a bivariate correlation analysis between HBV-DNA levels and cfDNA fluorescence intensities in these patients, and no correlation was found (r=0.015, P=0.863 for HBV-DNA and SE8; r=0.015, P=0.866 for HBV-DNA and SE37). This may be related to the fact that HBV-DNA in the blood is not free but present in the Dane particles (27).

After the aptamer was further added, the fluorescence intensity ascended continuously. Similarly, the corresponding original fluorescence indicators (SEA8 and SEA37) presented weak diagnostic value for PHC, but due to the fluorescence ascending less in PHC than LC, the related indicators SEA-SE8 and SEA-SE37 exhibited strong diagnostic value and were more valuable than cfDNA-related indicators. The change in fluorescence intensity after adding aptamers depends on the serum levels of free aptamers; that is, when more aptamers bind to targets, fewer free aptamers bind to the dyes, which results in weaker fluorescence intensity (28, 29). Thus, the smaller increase in fluorescence intensity in PHC than in LC suggests more specific targets of the aptamer in PHC serum than in LC serum.

The ATSFIs were measured at two temperature points, 8°C and 37°C. The serum fluorescence intensity was found to be stronger at 8°C than at 37°C, but the diagnostic performance was better at 37°C than at 8°C, and SEA-SE37 exhibited the greatest AUROC among all single fluorescence indicators. These results indicate that nonspecific binding at higher temperatures is reduced, which is a well-known phenomenon in biological binding reactions. Interestingly, the fluorescence indicators at the two temperature points were all independent variables in the diagnostic model, suggesting that they differently contribute to the diagnosis of PHC.

No significant differences were found between the aptamer-related fluorescence intensity indicator SEA-SE37 (similar in SEA-SE8) and the PHC subgroups of age, sex, Child-Pugh grade, tumor type, tumor size, BCLC stage, and TNM stage, suggesting that aptamer-related fluorescence intensity is independent of these clinicopathological characteristics of PHC. However, SEA-SE37 was significantly different between AFP-positive and AFP-negative PHCs, but its correlation with AFP was weak. These features give the indicator SEA-SE37 high diagnostic performance in various subtypes of PHC, including AFP-negative PHC, small PHC, and early-stage HCC, important for the translational application of the aptamer in clinical practice. The mechanism that SEA-SE was irrespective of PHC clinicopathological characteristics is unclear. We speculate that this may be related to a “combination detection” effect. The aptamer was generated using pooled serum and thus may target multiple types of molecules due to the extremely complex of serum components. Therefore, in the reaction between aptamer and serum, the aptamer binds to multiple targets simultaneously, similar to the combined detection of multiple indicators that yields better performance than a single indicator. Additionally, the targets of the aptamer may be irrespective of tumor size and stage of PHC. However, these speculations have yet to be confirmed.

We previously reported that the combination of serum autofluorescence and cfDNA fluorescence intensities had moderate diagnostic value for differentiating PHC from LC (21). In the present study, we combined aptamer-related fluorescence intensity with autofluorescence and cfDNA fluorescence intensities (“triple” instead of “dual”) and obtained robust diagnostic performance. The ATSFIs reflect the characteristics of PHC serum in three dimensions: autofluorescence, cfDNA-related fluorescence and aptamer-related fluorescence. Serum autofluorescence intensity has a certain correlation with malignant tumors (14). Serum cfDNA is well known to be associated with tumors and their levels can be analyzed by fluorescence (15). The aptamer specifically binds to targets, and the bound status between aptamers and targets can be analyzed by fluorescence (30). Therefore, the combination of the three dimensions in a logistic diagnostic model was able to provide powerful diagnostic ability in the present study. This novel concept could be transformed to other tumor diagnoses if a specific aptamer is available.

Although the aptamer-related fluorescence intensity (SEA-SE37) exhibited high diagnostic performance for PHC, even better than AFP, we did not know the target molecules of the aptamer. Therefore, we tried to realize the underlying mechanism of the aptamer for the diagnosis of PHC from clinical aspects. The SEA-SE37 was significantly correlated with many blood laboratory indicators with low or moderate correlation coefficients (absolute r=0.219-0.420) (**Table 3**), while some conventional laboratory blood tests have been proven to be valuable for the diagnosis of PHC in our studies (21, 31, 32) and others (33, 34), particularly in combination. Furthermore, SEA-SE37 was independent of the clinicopathological characteristics of PHC (**Figure 7**). The weak or no associations of aptamer-related fluorescence intensity with conventional laboratory blood tests and clinicopathological indicators may indicate that the aptamer is generally independent of single clinical and pathological factors but dependent on comprehensive characteristics of PHC serum, which makes SEA-SE37 present better diagnostic performance than AFP.

The clinical profiles of the PHC and LC groups differed significantly (**Table 1**). To realize whether the diagnostic performance of ATSFIs was related to the difference in clinical profiles, we performed propensity score matching (PSM) for PHC and LC using age, sex, etiology, Child-Pugh grade, and liver function indicators, and 150 pairs were successfully matched (**Supplemental Table S1**). In the matched set, SEA-SE37 remained the single indicator with the best diagnostic value and had an AUROC comparable to that before matching (0.858 vs. 0.879) (**Supplemental Figures S1A, B**), and the AUROC of the ATSFI model was also comparable to that before matching (0.917 vs. 0.937) (**Supplemental Figures S1C, D**). In addition, the pre-match AFSFI model exhibited an AUROC of 0.915 in the matched set (**Supplemental Figures S1E**). These results suggest that diagnostic performance is similar before and after PSM and virtually unaffected by the clinical profiles.

In the differences of the clinical profile, higher ALB levels and lower TBIL levels were found in the PHC group compared to the LC group (contradicting the tendency of PHC to occur in advanced fibrosis) (**Table 1**). This was due to the different liver function status between PHC and LC in the present study. PHC patients were hospitalized mainly for surgery and other antitumor treatments, which usually require liver function to be compensated. In contrast, LC patients were hospitalized mainly because of deteriorating or decompensated liver function. Therefore, the proportion of Child-Pugh grade A was significantly greater in the PHC group than in the LC group, while the opposite was for grades B and C (**Table 1**).

In the present study, we did not collect non-LC patients as controls, including chronic hepatitis and normal controls, due to the following considerations. Firstly, our new method is necessary to compare with the best current biomarker AFP, and serum AFP levels are usually elevated in the patients with chronic hepatitis hospitalized at our hospital due to active or severe disease, which may therefore lead to bias in this comparison. Secondly, from a clinical point of view, the differential diagnosis between liver cancer and cirrhosis is more important than that between liver cancer and hepatitis, because majority of PHC occurred in the background of cirrhosis rather than hepatitis. Thirdly, our previous study found that the grayscale value of aptamer-serum binding bands between cirrhosis and chronic hepatitis were very close (10), suggesting that cirrhosis may be “representative” of chronic hepatitis to some extent. As for the normal control, this is a “case-control” diagnostic study that tends to exaggerate diagnostic effects according to the STARD guidelines (35). However, the above-mentioned control groups should still be considered in the future for systematic evaluation of our new diagnostic method.

Additionally, further studies in other aspects will help us to better understand the ATSFI assay for the diagnosis of PHC. Validation studies in external patient cohorts can further evaluate the diagnostic performances of the ATSFIs for PHC. The etiology of patients in the present study was predominantly hepatitis B virus; thus, the diagnostic performance of the ATSFI method should be evaluated in PHC patients caused by other factors. Studies on the target molecules of the aptamer are helpful to elucidate the mechanisms underlying aptamer-based diagnosis.

In conclusion, a novel, feasible and robust diagnostic method for PHC has been successfully developed and internally validated. This method was established on ATSFIs that were sequentially measured in one tube by a conventional real-time PCR system, with the advantages of high throughput, rapidity, convenience, and low cost. ATSFIs were excellent in the diagnosis of PHC, including AFP-negative, early-stage and small PHCs, independent of and complementary to AFP. The excellent diagnostic performance of the ATSFI method is based on the comprehensive integration of triple serum fluorescence characteristics, especially aptamer-based fluorescence, and the independence of the clinicopathological characteristics of PHC. However, further studies that evaluate the diagnostic performance in external patient cohorts, analyze the exact targets of the aptamer, and explore the underlying diagnostic mechanisms will provide more information to understand the ATSFI method.

## Data Availability Statement

The datasets generated for this study are available on request to the corresponding authors.

## Ethics Statement

The studies involving human participants were reviewed and approved by The Ethics Committee on Medical Research of the First Affiliated Hospital of Nanchang University. The ethics committee waived the requirement of written informed consent for participation.

## Author Contributions

KZ and TW conceived and designed the study. JZ, FH, and HZ participated in the experiments. JZ, TW, and KZ analyzed and interpreted the data. SY and ZL collected clinical data. JZ, KZ, and TW prepared and revised the manuscript. All authors have read and approved the content of the final manuscript.

## Funding

This work was supported by the Natural Science Foundation of China (82160444, 81702107, 81760536) and the Science and Technology Project of Jiangxi Province, China (20171ACB21055 and 20192BBG70048).

## Conflict of Interest

The authors declare that the research was conducted in the absence of any commercial or financial relationships that could be construed as a potential conflict of interest.

## Publisher’s Note

All claims expressed in this article are solely those of the authors and do not necessarily represent those of their affiliated organizations, or those of the publisher, the editors and the reviewers. Any product that may be evaluated in this article, or claim that may be made by its manufacturer, is not guaranteed or endorsed by the publisher.
