# Supplementary material for: Aptamer-Based Triple Serum Fluorescence Intensity Assay: A Novel and Feasible Method for the Clinical Diagnosis of Primary Hepatic Carcinoma
Source: Front Oncol. 2022 Jun 7;12:897775. doi: 10.3389/fonc.2022.897775 (PMC9210211; doi:10.3389/fonc.2022.897775)

**Supplemental Table S1. Clinical profiles of patients matched by propensity score**

|  | **PHC (n = 150)** | **LC (n = 150)** | **P** |
| --- | --- | --- | --- |
| **Age (Mean ± SD, years)** | 53.3 ± 12.5 | 54.1 ± 12.1 | 0.565^a^ |
| **Sex [n (%)]** |  |  |  |
| Male | 111 (74.0) | 111 (74.0) | 1.000^b^ |
| Female | 39 (26.0) | 39 (26.0) |  |
| **Etiology [n (%)]** |  |  | 0.003^b^ |
| HBV | 125 (83.3) | 99 (66.0) |  |
| Non-HBV | 12 (8.0) | 25 (16.7) |  |
| Unknown | 13 (8.7) | 26 (17.3) |  |
| **Hepatic function test** |  |  |  |
| ALT (U/L) [M (IQR)] | 30.0 (22.0-49.0) | 26.0 (19.0-39.0) | 0.029^c^ |
| AST (U/L) [M (IQR)] | 39.0 (27.0-61.0) | 37.0 (29.0-56.0) | 0.717^c^ |
| TBIL (μmol/L) [M (IQR)] | 13.1 (9.3-19.2) | 16.1 (10.9-22.4) | 0.023^c^ |
| DBIL (μmol/L) [M (IQR)] | 4.5 (3.1-8.6) | 7.3 (4.6-10.2) | <0.001^c^ |
| GGT (U/L) [M (IQR)] | 70.5 (33.0-124.0) | 37.0 (21.0-82.0) | <0.001^c^ |
| ALP (U/L) [M (IQR)] | 109.0 (81.0-149.0) | 101.5 (78.0-143.0) | 0.192^c^ |
| TP (g/L) (Mean ± SD) | 66.1 ± 6.9 | 66.2 ± 7.2 | 0.896^a^ |
| ALB (g/L) (Mean ± SD) | 36.7 ± 5.5 | 36.7 ± 4.9 | 0.973^a^ |
| GLB (g/L) (Mean ± SD) | 29.3 ± 6.4 | 29.4 ± 7.2 | 0.896^a^ |
| **Child-Pugh grade [n (%)]** |  |  | 0.728^b^ |
| A | 123 (82.0) | 126 (84.0) |  |
| B | 18 (12.0) | 18 (12.0) |  |
| C | 9 (6.0) | 6 (4.0) |  |

a: Student’s t-test; b: Pearson Chi-squared test; c: Mann-Whitney U test. M (IQR): median (interquartile range); PHC: primary hepatic carcinoma; LC: liver cirrhosis; HBV: hepatitis B virus; ALT: alanine aminotransaminase; AST: aspartate aminotransaminase; TBIL: total serum bilirubin; DBIL: direct serum bilirubin; GGT: gamma glutamyl transferase; ALP: alkaline phosphatase; TP: total serum protein; ALB: serum albumin; GLB: serum gamma-globulins.
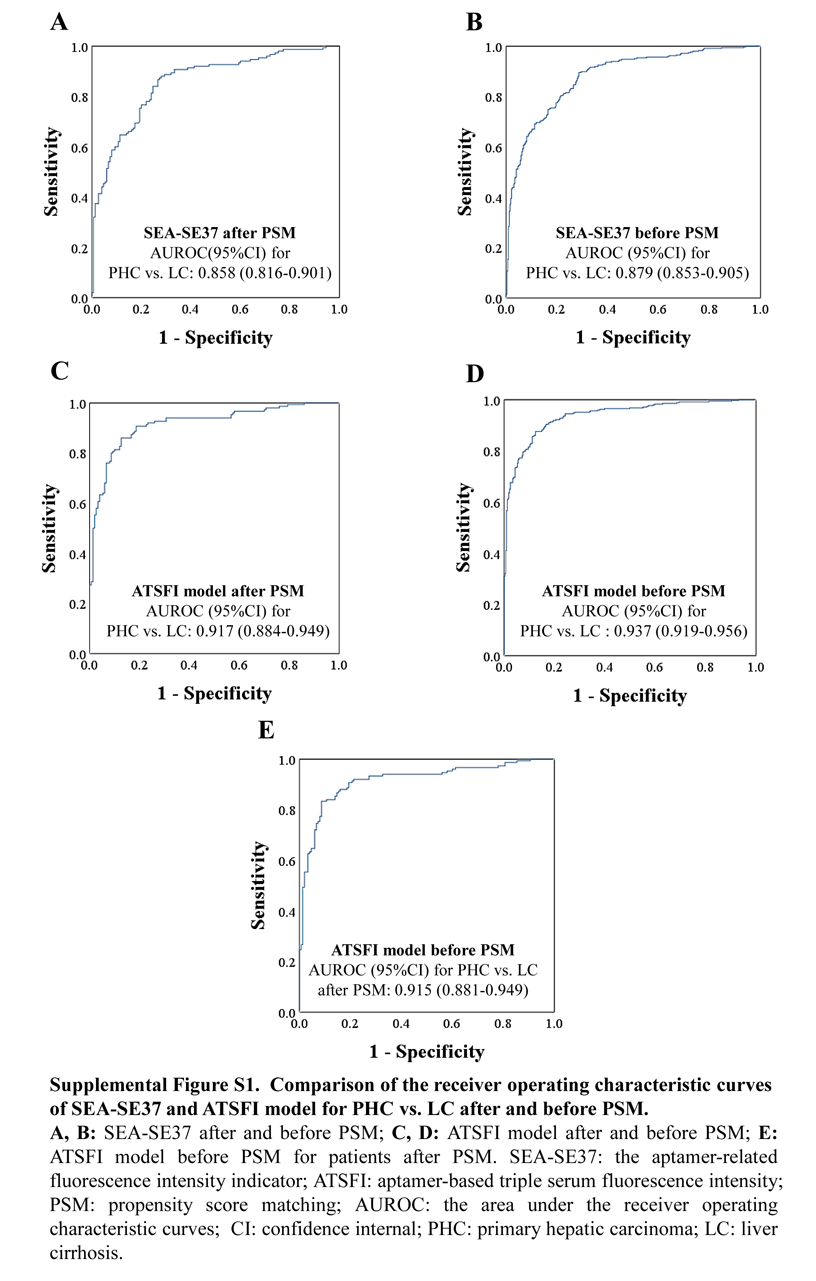

Supplement: Supplementary file 1 [file DataSheet_1.docx]
